# Sensory Impairments and Cardiovascular Disease Incidence and Mortality in Older British Community‐Dwelling Men: A 10‐Year Follow‐Up Study

**DOI:** 10.1111/jgs.13975

**Published:** 2016-02-18

**Authors:** Ann E. M. Liljas, S. Goya Wannamethee, Peter H. Whincup, Olia Papacosta, Kate Walters, Steve Iliffe, Lucy T. Lennon, Livia A. Carvalho, Sheena E. Ramsay

**Affiliations:** ^1^ Department of Primary Care and Population HealthUniversity College LondonLondonUK; ^2^ Population Health Research CentreDivision of Population Health Sciences and EducationSt George's University of LondonLondonUK; ^3^ Research Department of Epidemiology and Public HealthUniversity College LondonLondonUK

*To the Editor*: Hearing and vision impairments are common in older age. Evidence suggests that these sensory impairments are associated with incident cardiovascular disease (CVD) (myocardial infarction (MI), stroke),^1^ but previous studies have been undertaken mostly in specific subgroups of individuals with sudden sensorineural hearing loss or with stroke and in middle‐aged populations rather than community‐dwelling older adults.^2^, ^3^, ^4^, ^5^, ^6^, ^7^ Therefore, the association between self‐reported hearing and vision impairment and incident CVD, MI, and stroke and CVD mortality was examined in older men.

## Methods

Community‐dwelling men aged 63 to 85 (N = 3,981, 82% of the British Regional Heart Study cohort alive in 2003) were followed for 10 years, until 2013.^8^ Information on lifestyle factors, comorbidities, hearing, and vision was obtained through postal questionnaires. Self‐reported hearing aid use and ability to hear the television at a volume others find acceptable allowed for four categories of hearing: could hear (n = 2,851), could hear and used aid (n = 482), could not hear and no aid (n = 424), and could not hear and used aid (n = 168). Vision impairment was defined as not being able to recognize a friend across the street (n = 124). Dual sensory impairment (n = 57) consisted of hearing impairment (could hear with aid, could not hear and no aid, could not hear and used aid) and vision impairment. Follow‐up for CVD (nonfatal and fatal) was through general practice records and mortality registers. Survival analysis was used to examine the association between sensory impairments and incident CVD and mortality. Cox proportional hazards regression was used to calculate hazard ratios (HRs) with 95% confidence intervals (CIs) using no hearing impairment and no vision impairment (individually and combined) as reference groups. Prevalent CVD cases were excluded.

## Results

During the 10‐year follow‐up, 1,463 deaths occurred, including 408 CVD deaths. In 3,466 men free of prevalent CVD, 489 CVD events, 288 MIs, and 216 strokes occurred during follow‐up. In age‐adjusted analyses, men who could not hear and did not use a hearing aid had greater risks of incident CVD, incident stroke, and CVD mortality compared to men who could hear (Table 1). These associations remained statistically significant after adjustment for social class, diabetes mellitus, hypertension, obesity, smoking, and physical activity. The adjusted hazards ratio (95% CI) were 1.50 (1.14–1.98), 1.56 (1.04–2.34), and 1.39 (1.00–1.93) for incident CVD, stroke, and CVD mortality, respectively. These associations remained statistically significant after adjustment for social class, diabetes mellitus, hypertension, obesity, smoking, and physical activity. Vision impairment and dual sensory impairment were not associated with CVD incidence or CVD mortality.

**Table 1:** Risk of Outcome According to Sensory Impairment in Men Aged 63 to 85 in 2003 from the British Regional Heart Study

Sensory Impairment	Incident CVD		Incident Myocardial Infarction		Incident Stroke		CVD Mortality	
	Rate/1,000 (n)	HR (95% CI)	Rate/1,000 (n)	HR (95% CI)	Rate/1,000 (n)	HR (95% CI)	Rate/1,000 (n)	HR (95% CI)
Hearing								
Could hear	17 (330)	1.00	9 (191)	1.00	7 (149)	1.00	10 (257)	1.00
Could hear, used aid	20 (59)	0.91 (0.68–1.20)	13 (40)	1.09 (0.77–1.55)	7 (23)	0.76 (0.49–1.19)	17 (68)	1.15 (0.88–1.51)
Could not hear, no aid	25 (69)	1.42 (1.09–1.84)^a^	13 (38)	1.35 (0.95–1.91)	11 (32)	1.46 (1.00–2.14)^a^	15 (52)	1.37 (1.02–1.85)^a^
Could not hear, used aid	22 (22)	1.10 (0.71–1.70)	14 (14)	1.26 (0.73–2.17)	8 (8)	0.88 (0.43–1.80)	15 (20)	1.11 (0.71–1.76)
Vision								
Could see	18 (467)	1.00	10 (273)	1.00	8 (209)	1.00	12 (383)	1.00
Poor vision	24 (16)	1.20 (0.73–1.97)	16 (11)	1.41 (0.77–2.57)	7 (5)	0.85 (0.35–2.06)	19 (17)	1.42 (0.87–2.30)
Dual								
Could hear and could see	17 (326)	1.00	9 (185)	1.00	8 (151)	1.00	10 (254)	1.00
Dual impairment	26 (8)	1.40 (0.69–2.83)	13 (4)	1.23 (0.46–3.31)	13 (4)	1.52 (0.56–4.12)	22 (9)	1.73 (0.89–3.36)

a Remained statistically significant after further adjustment for social class, obesity, smoking, physical activity, hypertension, and diabetes mellitus.

CVD = cardiovascular disease; HR = hazard ratio; CI = confidence interval.

## Discussion

Men who could not hear and did not use a hearing aid had greater risks of incident CVD, particularly incident stroke, and CVD mortality than men who could hear. Previous research suggests that the associations between hearing impairment and CVD could be attributed to smoking and atherosclerosis,^9^ but in the current study, the associations remained significant after adjustment for smoking and CVD‐related comorbidities. Not all hearing impairment groups were associated with CVD incidence, suggesting that hearing per se may not underlie the observed associations. One possible mechanism could be cognitive impairment, which is related to hearing impairment and stroke.^10^ Other possible explanations could be atherosclerotic or inflammatory changes, which could not be taken into account in the analyses.^9^ Moreover, hearing impairment based on self‐report could be subject to inaccurate reporting of hearing impairment because of unawareness, denial of hearing problems, or use of hearing aid. Any inaccurate reporting may have underestimated the influence of hearing impairment on CVD and may also explain the inconsistent associations between the hearing impairment groups. Although the findings are consistent with those of earlier studies that found objectively measured hearing impairment to be associated with incident stroke,^2^, ^3^ and CVD mortality,^1^ another study found no association between objective hearing impairment and incident stroke.^5^ These inconsistent findings could be due to different pathologies that underlie different types of hearing impairment, such as sensorineural (sudden) or age‐related (gradual) hearing loss.

The lack of association between vision impairment and incident CVD and CVD mortality could be due to the definition of vision impairment used, which may have identified severe vision impairment only, thus underestimating the true prevalence of vision impairment. Similarly, dual sensory impairment was not associated with CVD incidence or mortality, which could be due to the small number of men with dual sensory impairment.

## Conclusions

Hearing impairment in older men was associated with greater risks of incident stroke and CVD mortality. Early detection of hearing impairment in older adults could help prevent CVD. Further research is warranted into the possible mechanisms underlying these associations.
